# Primary gliosarcoma with widespread extracranial metastases—spatiotemporal morphological variation

**DOI:** 10.1186/s41016-022-00285-1

**Published:** 2022-08-05

**Authors:** Ming Luo, Jun Yang, Jianjun Sun, Fengyun Wang, Xiaodong Chai

**Affiliations:** 1 Medical Imaging Department, Third Affiliated Hospital of Baotou Medical College, Baotou, Inner Mongolia, China; 2 Department of Neurosurgery, Peking University Third Hospital, Beijing, China; 3 Department of Oncology, First Affiliated Hospital of Baotou Medical College, Baotou, Inner Mongolia, China; 4 Department of Pathology, Peking University Third Hospital, Beijing, China

**Keywords:** Brain gliosarcomas, Extracranial metastasis, Spatiotemporal morphological variation

## Abstract

**Background:**

We summarize 5 cases of primary gliosarcoma with widespread extracranial metastases including our case. The glial components are eliminated due to the needs of the living environment in the process of parasitism and survival of brain glioma-sarcoma cells in lung metastasis.

**Methods:**

A PubMed search using the keywords “gliosarcoma” and “extracranial metastases” was performed followed by a review of cited literature. Our case was a 50-year-old female presented with headache and dizziness. MRI examination showed that there was a cystic solid tumor in the right temporal lobe. The tumor was removed totally. Seven months after the operation, the patient suffered recurrent intermittent headache. The resection for the recurrent tumor was performed. Postoperative pathology confirmed the recurrent gliosarcoma. A needle biopsy was performed for the nodular on the right lung. The lung tumor pathology suggested a sarcoma structure.

**Results:**

There was a female patient in five cases. The age range is 47 to 69 years old. The tumor recurred within a year. A combination of treatment modalities may extend survival; however, the prognosis remains poor.

**Conclusion:**

Primary gliosarcoma with extracranial metastases is extremely rare. Some findings uncovered an unexpected spatiotemporal morphological variation in the different foci of the same malignancy.

## Background

Gliosarcoma is a rare central nervous system tumor, a variant of glioblastoma. In spite of the active comprehensive treatment in clinical practice, glioma is still a kind of intracranial malignant tumor that is difficult for clinicians to deal with and has a poor prognosis. At present, surgical resection is regarded as the first choice for the treatment of glioma. However, whether postoperative adjuvant radiotherapy and chemotherapy can improve its prognosis, and which other factors may be related to the prognosis, have not yet reached a consensus. In previous reports on glioma, the probability of systemic metastasis was low. We summarized 5 cases of primary gliosarcoma with widespread extracranial metastases including our case (Table 1).

**Table 1:** Literature review of primary gliosarcoma with widespread extracranial metastases

Author, year, reference	Age Years	Sex	Clinical presentation	Management	Outcome
Min Gyu Choi et al. Brain Tumor Res Treat 2020 [1]	69	M	Headache, mild dizziness, and left-side weakness	Surgery + RT + CT	Died 9 months
Atef Ben Nsir et al. World Neurosurg 2015 [2]	57	M	Chest pain, headaches, and vomiting	Surgery + RT + CT	Doing well at 1 year
Marion Rapp. Br J Neurosurg 2011 [3]	67	M	General seizures	Surgery + RT + CT	Died 12 months
Thomas L. Beaumont et al. J Neurooncol 2007 [4]	47	M	Headaches with weakness	Surgery + ^125^I + RT + CT	Died 19.5 months

## Methods

1. A PUBMED search using the key words “gliosarcoma” and “extracranial metastases” was performed followed by a review of cited literature. We retrieved four articles (Table 1).
2. Our case was a 50-year-old female presented with headache, dizziness. MRI examination showed that there was a cystic solid tumor in the right temporal lobe. The tumor was removed totally. She received standard- dose radiation therapy and multiple courses of temozolomide chemotherapy after the operation. Seven months after the operation, the patient suffered recurrent intermittent headache. The resection for the recurrent tumor was performed. Postoperative pathology confirmed the recurrent gliasarcoma. A needle biopsy was performed for the nodular on the right lung. The lung tumor pathology suggested a sarcoma structure. The patient died 24 months after metastasis.

### Case 1

A 69-year-old male presented with a week of worsening headache, mild dizziness, and left-side weakness. The right frontal lobe tumor was totally resected. He underwent adjuvant radiotherapy with concurrent temozolomide. Approximately, 7 months after surgery, the patient complained of epigastric pains. Positron emission tomography-CT scans revealed the right lung, left pleura, liver, lymph nodes, bones, and muscles. Liver biopsy revealed typical gliomatous and sarcomatous components. The patient was therefore diagnosed with metastatic gliosarcoma who died 9 months after the diagnosis of primary gliosarcoma. The patient’s initial histopathologic diagnosis was WHO grade IV, isocitrate dehydrogenase (IDH)-wild type. Histopathological analysis of the brain tumor reveals high cellularity as well as cellular and nuclear anaplasia. Image of glial fibrillary acidic protein (GFAP) staining shows the gliomatous component of the tumor.

### Case 2

A 57-year-old man presented with a 3-month history of chest pain, weight loss, headaches, and vomiting. The radiological work-up revealed 6 × 6 cm right apical pulmonary tumor and a 4 × 3.5 × 3.8 cm peripherally enhancing left cerebellar mass. Cerebellar mass was removed. The microscopic features and the immunohistochemical profile confirmed the diagnosis of gliosarcoma. The thoracic lesion was removed subsequently. Pathology confirmed it was an extracranial metastasis from the cerebellar gliosarcoma. Adjuvant radiation and chemotherapy were then administered. No clinical or radiographic evidence of recurrence was observed during 1 year of follow-up monitoring. The tumor displayed a biphasic pattern with areas of gliomatous and sarcomatous differentiation. The gliomatous component consisted of atypical glial cells with pleomorphic nuclei and frequent mitotic figures. The tumor predominantly consisted of spindle cell proliferation forming sarcomatous areas with intersecting fascicular or storiform patterns similar to fibrosarcoma. The gliomatous component showed immunoreactivity for glial fibrillary acid protein (GFAP), in contrast with the lack of immunoreactivity for glial fibrillary acid protein in the sarcomatous component.

### Case 3

A 67-year-old male patient presented with general seizures. MRI demonstrated a left temporo-occipital cystic, contrast-enhancing tumor. A left posterotemporal craniotomy with fluorescence-guided resection was performed. Histological investigation revealed a gliosarcoma. Three weeks following surgery, radiotherapy and concomitant chemotherapy with temozolomide were initiated. Immunohistochemistry revealed expression of glial fibrillary acidic protein (GFAP) and MAP2. Immunoreactivity for vimentin and protein S100 as well as nuclear positivity for the p53 tumor suppressor protein was detected in both the glial and the chondrosarcomatous tumor areas. Biopsy specimens in lung metastasis revealed small fragments of acellular tumor with partial chondrosarcomatous differentiation. Immunohistochemical investigation showed focal expression of MAP2 in tumor cells. Both MAP2-positive glial cells and chondrosarcomatous cells demonstrated immunopositivity for protein S100 as well as nuclear p53 accumulation.

### Case 4

A 47-year-old male presented progressively worsening headaches with weakness and numbness of the left extremities culminating in a fall. Postmortem histologic examination revealed widespread infiltrating gliosarcoma. The general postmortem examination demonstrated numerous nodules of metastatic sarcoma involving the scalp adjacent to the craniotomy site, the lip mucosa, chest wall, pleura, lungs, pericardium, myocardium, peritoneum, diaphragm, liver, pancreas, gastric mucosa, spleen, thyroid gland, and kidney. Tumor emboli were abundant, particularly in the spleen, liver, kidney, and lung. The glial component showed strong immunoreactivity to glial fibrillary acidic protein (GFAP) and S-100. The sarcomatous component was unstained with GFAP or S-100 but showed immunoreactivity to vimentin.

### Case 5

**Fig. 1:**
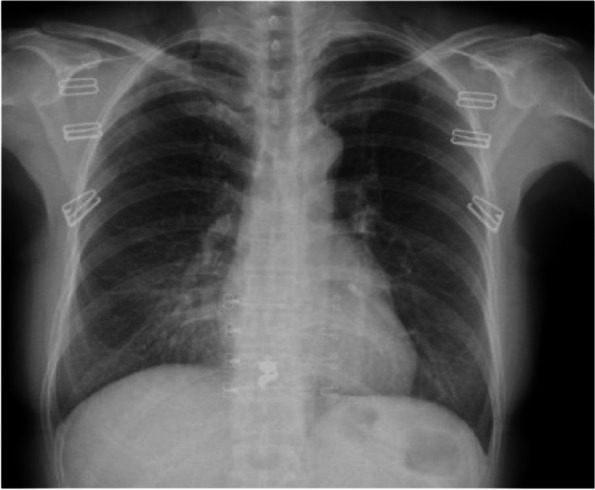
There were no abnormal nodules or space occupying in the lungs on the chest X-ray before first operation

**Fig. 2:**
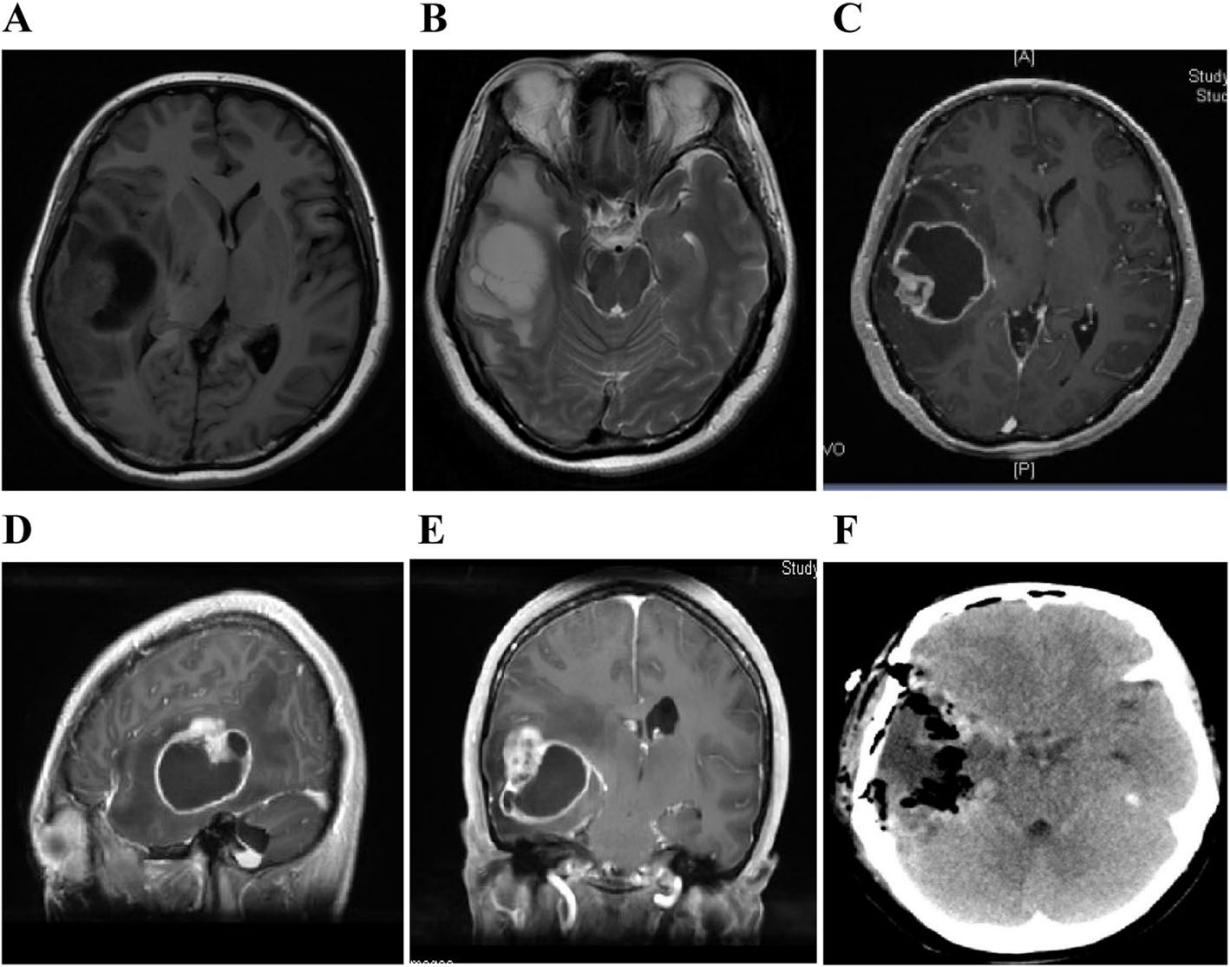
The first preoperative magnetic resonance images of the patient showed a solid cystic space in the right temporal lobe. **A** T1 phase showed a long and equal T1 signal occupying lesions in the right temporal brain, and the midline was squeezed and shifted to the left. **B** T2 phase showed a long T2 signal shadow that was larger than the actual occupying lesion. The edema was significant around the lesion. **C** Axial enhancement. **D** Sagittal enhancement. **E** Coronal enhancement all showed solid cystic tumor in the right temporal brain, nodular enhancement of the solid part, and ring enhancement of cystic lesions. **F** The postoperative CT showed that the tumor was completely removed, without bleeding in the local surgical field, and midline shift was restored

A 50-year-old female presented with headache, dizziness without incentives in May 2018, these symptoms could be alleviated by rest. The patient felt her symptoms worsened 1 week ago, and her headache was not significantly relieved after rest. He went to a hospital for a head MRI examination and showed that there was a cystic solid tumor in the right temporal lobe, which was considered to be highly malignant. There was no abnormality in the specialist physical examination (Figs. 1 and 2). The right temporal tumor was removed totally under general anesthesia on June 4, 2018. The process of operation was smooth, and no obvious residual symptoms were seen.

**Fig. 3:**
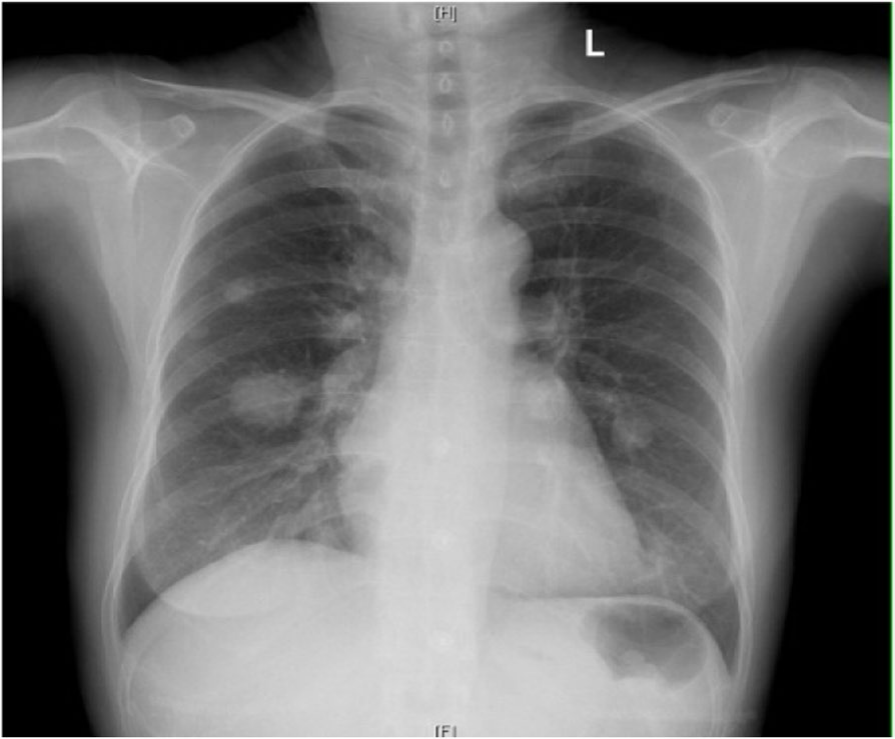
After the second admission, the preoperative chest radiograph showed nodular space in the right lung

**Fig. 4:**
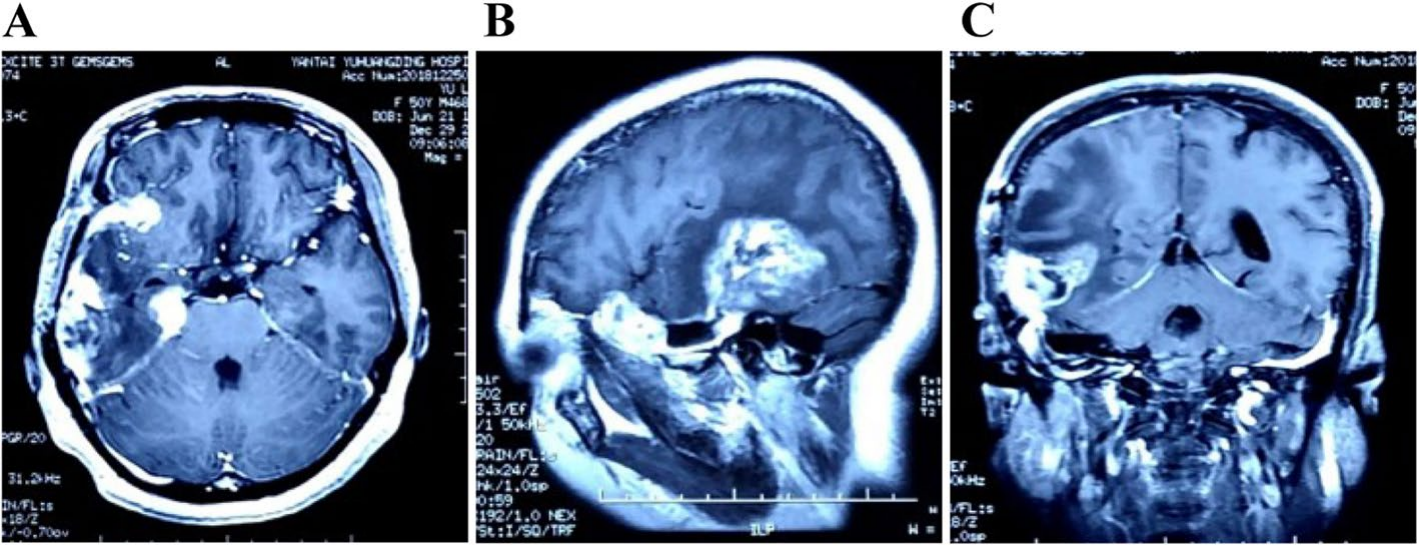
After the second admission, the preoperative MRI scan showed the recurrent tumor in the right temporal lobe. Multiple enhanced nodes had been spread to frontal lobe and extended to subtemporal area. **A** Enhanced axial scan. **B** Enhanced sagittal scan. **C** Enhanced coronal scan

In January 2019, the patient suffered recurrent intermittent headache without obvious cause, accompanied by slight dizziness, which could be relieved after rest. There was no abnormality in the sensory and movement of the limbs, without severe disturbance of consciousness. After being infused with mannitol and hormone in the local hospital, her symptoms resolved. A re-examination of the brain MRI showed that recurrent tumor with the abnormal enhancement in the right temporal lobe is progressing more than before (Figs. 3 and 4).

**Fig. 5:**
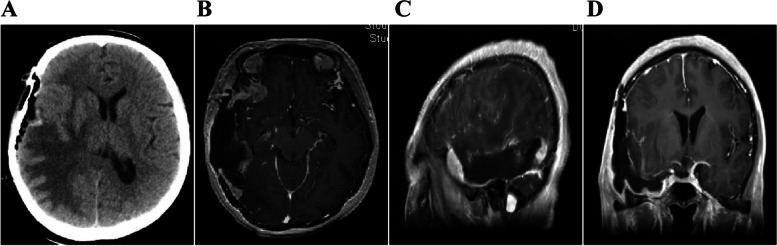
The second craniotomy was performed for the recurrent intracerebral tumor. During the operation, it was found that the reddish-brown supply rich tumor tissue broke through the dura mater and grew deep into the temporal muscle. **A** Postoperative CT showed that the recurrent tumor was removed, and the surgical field was clean without bleeding. **B** Enhanced axial view. **C** Enhanced sagittal view showed that there was still tumor tissue remaining in the frontal lobe. **D** Enhanced coronal view showed that the recurrent temporal lobe tumor was completely removed

**Fig. 6:**
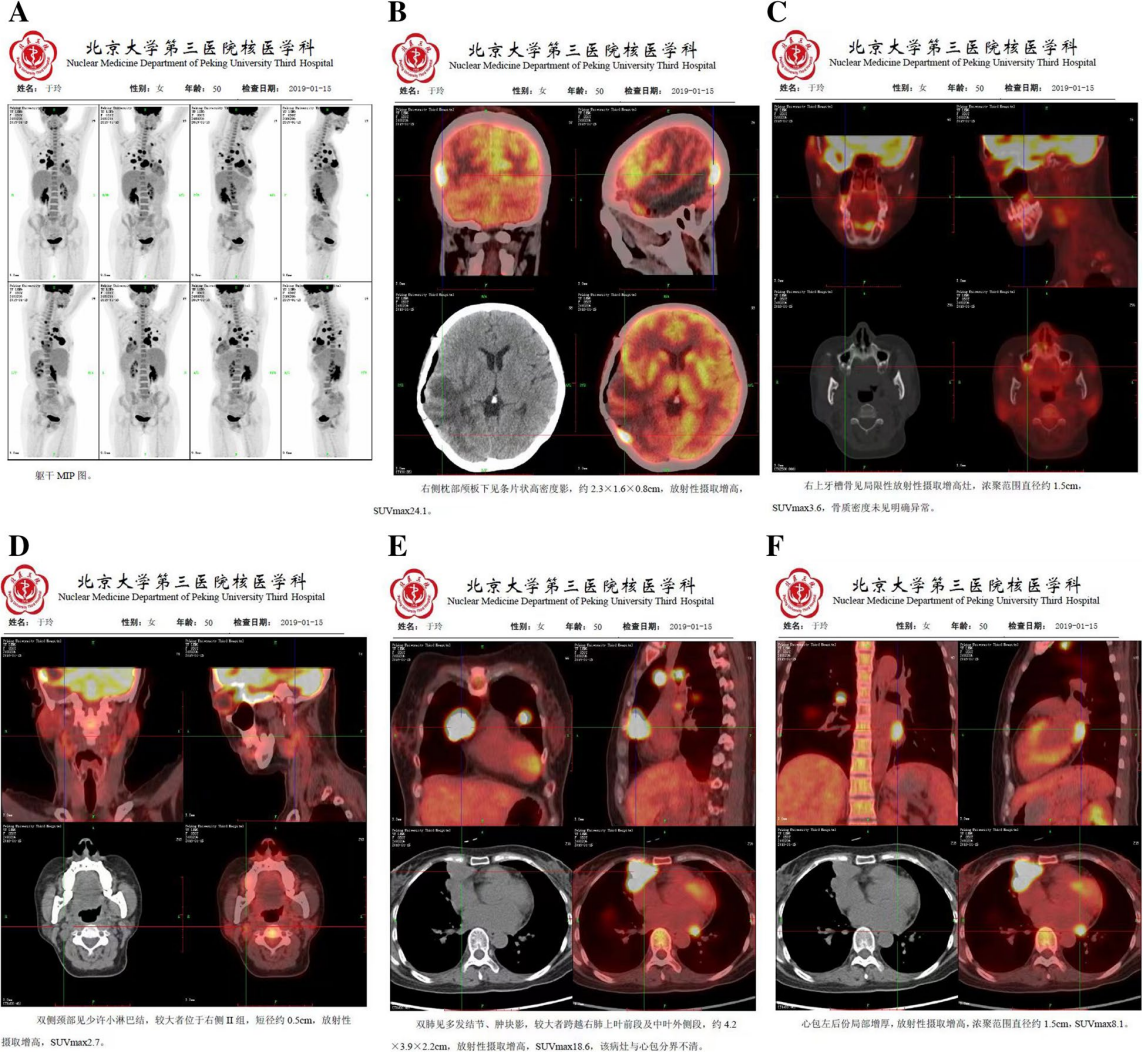
The recurrent gliosarcoma in the right temporal lobe, a whole body PET-CT was performed. **A** Multiple lesions dissemination and metastasis throughout the body. **B** Adjacent to the right temporal sublaminar residual lesions, multiple dissemination lesions were showed under the right occipital and frontal skull. **C** Suspicious metastatic lesions in the right mandible. **D** Suspicious metastatic lesions in the axis vertebral body. **E** Multiple metastatic nodular lesions in both lungs (subsequent pulmonary nodule biopsy was confirmed to be possible spread of gliosarcoma). **F** Involving the pericardium

The resection for the recurrent tumor was performed under general anesthesia in January 9, 2019. Postoperative pathology confirmed the recurrent gliosarcoma (Fig. 5). Whole body PET-CT was performed on January 15, 2019. The residual gliosarcoma was shown adjacent to the sublaminar hypermetabolism and intracranial multiple metabolically active lesions shown on the right occipital and frontal. Multiple nodular lesions involved in both lungs, in the left posterior part of the pericardium, and in lymph nodes of right hilar; all of the above lesions were considered as metastatic gliosarcoma (Fig. 6).

**Fig. 7:**
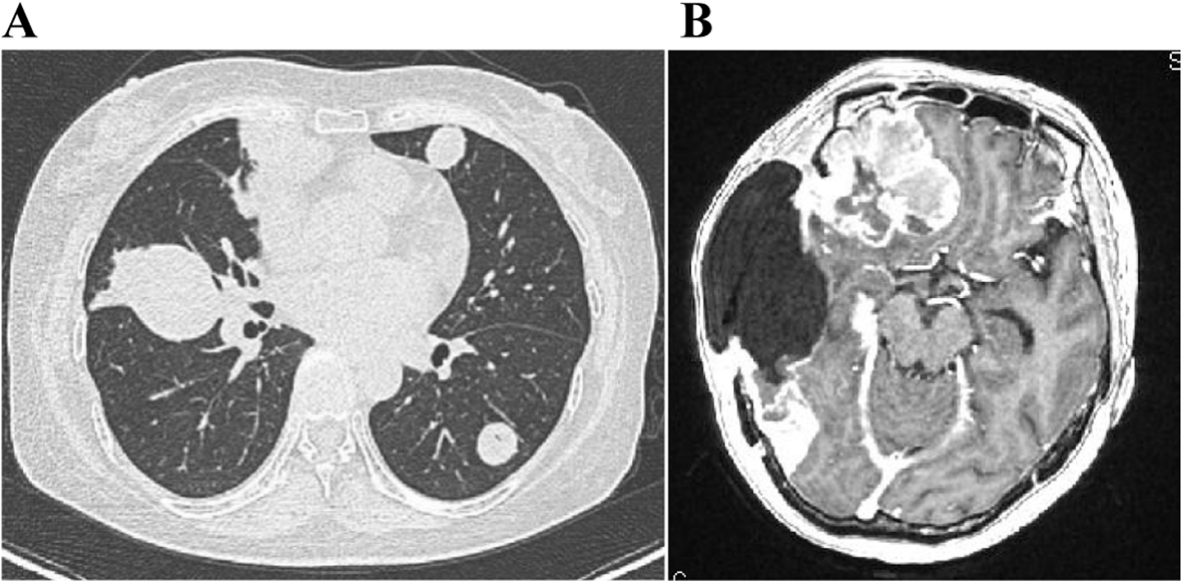
One month after the second craniotomy and puncture biopsy of the lung lesions, **A** re-examination of the chest radiograph showed enlarged lung metastases. **B** Intracranial right frontal lobe and occipital lobe lesions grew rapidly and appeared extensive meningeal metastasis

Multiple and active proliferating nodulars were confirmed by chest CT scans on January 25, 2019. A needle biopsy was performed for the nodular on the right lung. The lung tumor pathology suggested a sarcoma structure. The two tumor tissues were compared with genetic testing. The histomorphology and gene phenotype were consistent, indicating that the recurrent gliosarcoma and lung sarcoma were of the same source (Tables 2, 3, 4 and 5). Brain gliosarcoma may be directly spread to the lungs and then blood metastases to the whole body, but not spread by cerebrospinal fluid. However, in the process of parasitism and survival of brain glioma sarcoma cells in lung tissues, the glial components are eliminated due to the accommodation for surrounding living environment. After the second operation, brain MRI scan was checked again. The residual and metastasized frontal and occipital gliosarcomas grew and proliferated rapidly again (Fig. 7).

**Table 2:** Pathological results and immunohistochemistry analysis of two operation

Operation time	Pathology	Immunohistochemistry
04 June 2018	(Intracranial space). Malignant tumors, except for gliosarcoma	ATRX ( +), P53 (mutant), IDH1R132H ( −), Ki-67 (40% +), Oligo-2 ( +), GFAP ( +)
19 Jan. 2019	(Right temporal lobe mass). Glioblastoma, WHO grade V, most of the tumor cells are spindle-shaped, some are epithelioid, some are obviously atypia, and there are tumor giant cells with obvious necrosis. Combined with the medical history, it is consistent with the recurrence of gliosarcoma	GFAP(+), ATRX(+), SMA(+), EMA(+), P53(mutation), IDHR132H(-), S-10(+)0, KI-67(20%-30%)

**Table 3:** Results of lung nodule biopsy and immunohistochemistry analysis

Lung puncture time	Pathology	Immunohistochemistry
25 Jan. 2019	(Lung mass and soft tissue). Malignant tumors can be seen in the puncture tissues submitted for examination. Most of the tumor cells are fusiform, and some are epithelioid. Considering the history of the disease, the possibility of metastatic tumors is high. Pulmonary sarcomatoid carcinoma is not excluded. Supplementary diagnosis: (lung tumor soft tissue 1) (lung tumor soft tissue 2) malignant tumors can be seen in the submitted puncture tissue. Most of the tumor cells are fusiform, and some are epithelioid. Combined with the results of immunohistochemistry, they tend to be carcinosarcoma	CK mixed (individually weak +), P40 ( −) TTF-1 (minority +), Ki-67 (40% +), Vimentin ( +), CK7 (minority +), Napsin A ( −), GFAP ( −), ATRX( +), P53 (mutant), S-100 ( −) WT-1 ( −). Oligo-2 ( −) Note: This patient has a history of intracranial glioma, but there is no typical component of glioma in the lung tumor puncture tissue. Since the extracranial metastasis of glioma is very rare, it is recommended that both sites are primary tumors. Further detection of P53 germline mutations to exclude the possibility of Li-Fraumeni syndrome

**Table 4:** Accurate gene detection report of recurrent gliomas (21 Jan. 2019)

Result	Gene				
Wild type	IDH1 R132 point mutation	**IDH2 R172 point mutation**	H3F3A K27M point mutation	**HIST1H3B K27M point mutation**	BRAF V600E point mutation
Mutant	**TERT C228T/C250T point mutation, C228T mutant**				
No joint deletion	1p/19q combined deletion				
Negative	MGMT promoter methylation

**Table 5:** 19 gene detection of precise drugs for lung metastases (11 Jan. 2019)

Result	Gene								
Negative	AKT1	**ALK**	ARAF	**BRAF**	DDR2	**EGFR**	**ERBB2(HER2)**	FGFR1	FGFR3
	**KRAS**	MAP2K1	**MET**	NRAS	NTRK1	NTRK3	PIK3CA	**ROS1**	**RET**
Positive	PTEN								

The pathological result of the original brain tumor showed malignant tumor, but gliosarcoma needed to be confirmed. Immunohistochemical result indicated Tp53 mutation. GFAP staining was positive. Ki-67 staining was 40% positive, which were in accordance with glioma.

The pathological result of the recurrent right temporal tumor showed gliosarcoma, WHO grade V. Morphology showed many spindle-shaped tumor cells, some with epithelioid cells and some with distinctly heteromorphic. The giant gliosarcoma cells were consistent with obvious necrosis.

The biopsy tissues of lung neoplasm were malignant tumor cells, with most of the spindle, part of epithelioid. Morphology was the same with the recurrent gliosarcoma, considering possibility of metastatic tumor from brain gliosarcoma. Immunohistochemical result indicated that Tp53 was mutation, and Ki-67 was 40% positive, but GFAP was negative. The glial components are eliminated due to the accommodation for surrounding living environment.

The two tumor tissues were compared through genetic testing, and the histomorphology and gene phenotype were consistent, indicating that the recurring brain glioma and lung sarcoma were of the same source. This patient had short-term recurrence and distant metastasis after operation, which was consistent with the results of genetic analysis. This patient had a negative BRAF V600E, indicating that he could not benefit from BRAF inhibitors; a negative MGMT methylation indicated that the patient had a poor prognosis and could not benefit from temozolomide chemotherapy: IDH1, IDH2 negative, and 1P/19q heterozygous deletion negative, indicating a bad prognosis and insensitivity to radiotherapy and chemotherapy; and TERT mutation-positive, analyzed in the IDH wild-type subgroup, patients prognosis is worse.

## Discussion

Gliosarcoma accounts for 0.48% of all intracranial tumors and 2–8% of glioblastomas [5]. It occurs between 50 and 60 years old, and the ratio of male to female is 1.4 ~ 1.8:1 [6, 7]. The most common locations are temporal lobe, frontal lobe, parietal lobe, and occipital lobe [8]. The clinical features depend on the location of tumor and are similar to glioblastoma. The most common symptoms are headache, vomiting, epilepsy, hemiplegia, cognitive decline, and other symptoms related to increased intracranial pressure [6].

Imaging may appear as a central necrotic area like glioblastoma or with uniform enhancement and sharp edges like meningiomas. Histologically, two different cell populations can be identified; one is composed of astrocytes that meet the criteria for glioblastoma, and the other is composed of spindle cells of the sarcoma [9]. Malignant gliomas rarely metastasize to extracranial sites. As we all know, the metastatic ability of gliosarcoma is very strong, and its incidence rate can reach 11%, which is much higher than that of glioblastoma (0.2–1.2%) [10]. Metastasis may be related to the location of the temporal lobe, close to the dura mater and venous sinuses [11]. Dural infiltration and extracranial metastasis are more common in glioblastoma. The main sites of metastasis are lungs, liver, and lymph nodes [10]. Other reported sites are the spleen, adrenal glands, kidneys, oral mucosa, skin, bone marrow, skull, ribs, and spine. In young men who have received adjuvant radiotherapy, extracranial transfer is more common, which is related to a poor prognosis. Distant metastasis is mostly related to the recurrence of glioma. Beaumont et al. [12] reported a case of gliosarcoma with multiple extracranial metastases, and an intravascular tumor embolus was found on autopsy, which is consistent with a greater tendency to spread blood.

Recent theories believe that the monoclonal origin of glioblastoma and sarcoma components is formed by malignant glioma mesenchymal differentiation, which explains why there is no significant difference in clinical outcomes between glioblastoma and gliosarcoma [7]. Although there is no systematic epidemiological data to support this hypothesis, studies have shown that gliosarcoma is more likely to spread outside the skull compared with typical glioblastoma.

Gliosarcoma has been reported to have a higher rate of systemic metastasis than expected [13]. It differs from primary IDH wild-type glioblastoma by a slightly higher PTEN mutation rate and infrequent EGFR alterations [14]. Maria-Magdalena Georgescu et al. [15] reported a unique, complex case of recurrent glioblastoma with multifocality, multicentricity, and extraneural lung metastasis and noted two opposite genetic evolution patterns in the intraneural and extra-neural compartments. The majority of the viable tumor from the first resection exhibited the biphasic morphological and staining pattern of gliosarcoma, with mutually exclusive GFAP-positive glial and reticulin-positive sarcomatous areas.

The lung biopsy showed a heterogeneous tumor with diffuse GFAP expression, which exhibited areas of pleomorphic cells embedded in a myxoid extracellular matrix showing brisk mitotic activity and areas of fibroblastic-like cells with lower mitotic activity. Importantly, it showed a striking accumulation of mutations in the lung metastasis, leading to significantly increased TMB and strong activation of the PI3K/PTEN/AKT and p53 pathways, with critical pathogenic and therapeutic implications. These findings uncovered an unexpected spatiotemporal morphological variation in the different foci of the same malignancy.

Accumulation of a distinct mutation in TP53 has been previously reported in a metastatic glioblastoma case [16]. Sung-Yup Cho1 [17] found that TP53 mutations make it easier for cells to differentiate through tumor epithelial-mesenchymal cell-like processes. In addition, TP53 mutations played a vital role in the development of gliosarcoma through mesenchymal differentiation; our data suggests that TP53 mutations were also related with the resistance of treatment.

MET overexpression in the primary gliosarcoma and the lung metastasis may be responsible for the fibroblast-like morphology of these tumors and possibly the metastatic potential, as c-MET is a receptor tyrosine kinase involved in epithelial to mesenchymal transition, invasion, and metastasis [18]. Both MET and VEGFA are hypoxia-regulated genes through the activation of HIF-1α [19, 20]. These tumors were extensively necrotic, most likely mounting a strong anti-hypoxic response. The supratentorial recurrences adopted an epithelial morphology, and unknown environmental cues may have stimulated a reverse cellular mesenchymal to epithelial transition response.

Epidural malignant glioma metastasis is most common in patients whose iatrogenic surgery may touch extracerebral structures. Surgical removal of insufficient tumor borders, iatrogenic tumor rupture, and its spread through the surgical area, or implantation of tumor cells into remote areas through the use of contaminated surgical tools are considered to be the main causes of local and regional recurrence. The use of contaminated surgical tools to transplant highly malignant tumor cells was reported to be 87%. Animal experiments had shown that the number can rise up to 100% in malignant melanoma.

Therapeutic methods of gliosarcoma include tumor resection, postoperative radiotherapy, and chemotherapy [7]. According to reports, the median survival time of gliosarcoma is 9 months, but there are some changes [21]. The median survival time of patients diagnosed before the age of 50 is 15 months, while that after the age of 50 is 7 months. Radical resection can prolong survival for 7–11 months, while some simple biopsy is only 4 months. Radiation therapy improves survival from 4 to 10 months. The role of chemotherapy is still uncertain, but there are a few encouraging case reported.

Surgery is the first line of treatment for malignant glioma. Once there is metastasis, the ideal treatment is still unclear; regular chemotherapy does not seem benefit. At present, there is no standard treatment for the treatment of intracranial and extracranial metastases, and they are currently palliative. Although the mechanism of intracranial and extracranial metastases is still unclear, iatrogenic spread through surgery may be the main cause [22, 23]. Gliosarcoma forms metastases along the nerve transmission path or implants into the peritoneum through ventricular-peritoneal shunt [24–26]. In addition, radiotherapy can induce the transformation of glioblastoma to sarcomatoid metaplasia, which in turn forms the ability to penetrate the blood vessel wall [27]. Our reported case has a history of two craniotomy. Local radiotherapy and systemic chemotherapy were performed after surgery.

## Conclusion

In short, temporal glioma can spread to the lungs and other parts of the body outside the skull. The mechanism is not yet clear. It may be directly spread to the lungs and then blood metastases to the whole body, not necessarily spread by cerebrospinal fluid result. However, in the process of parasitism and survival of brain glioma sarcoma cells in lung tissues, the glial components are eliminated due to the needs of the living environment.

## Data Availability

Please contact the authors for data requests.
